# Whole transcriptome sequencing identifies key circRNAs, lncRNAs, and miRNAs regulating neurogenesis in developing mouse retina

**DOI:** 10.1186/s12864-021-08078-z

**Published:** 2021-10-30

**Authors:** Gang Chen, Hong-Mei Qian, Jing Chen, Jie Wang, Ji-Tian Guan, Zai-Long Chi

**Affiliations:** State Key Laboratory of Ophthalmology, Optometry and Visual Science, Eye Hospital of Wenzhou Medical University, Wenzhou, China

**Keywords:** Whole-transcriptome sequencing, circRNA, lncRNA, miRNA, Neurogenesis, Retina development, Bioinformatics analysis

## Abstract

**Background:**

The molecular complexity of neural retina development remains poorly studied. Knowledge of retinal neurogenesis regulation sheds light on retinal degeneration therapy exploration. Therefore, we integrated the time-series circRNA, lncRNA, miRNA, and mRNA expression profiles of the developing retina through whole-transcriptome sequencing. The key functional ncRNAs and the ceRNA network regulating retinal neurogenesis were identified.

**Results:**

Transcriptomic analysis identified circRNA as the most variable ncRNA subtype. We screened a series of neurogenesis-related circRNAs, lncRNAs, and miRNAs using different strategies based on their diversified molecular functions. The expression of circCDYL, circATXN1, circDYM, circPRGRIP, lncRNA Meg3, and lncRNA Vax2os was validated by quantitative real-time PCR. These circRNAs and lncRNAs participate in neurotransmitter transport and multicellular organism growth through the intricate circRNA/lncRNA-miRNA-mRNA network.

**Conclusion:**

Whole-transcriptome sequencing and bioinformatics analysis systematically screened key ncRNAs in retinal neurogenesis. The validated ncRNAs and their circRNA/lncRNA-miRNA-mRNA network involve neurotransmitter transport and multicellular organism growth during retinal development.

**Supplementary Information:**

The online version contains supplementary material available at 10.1186/s12864-021-08078-z.

## Background

As an essential part of the central nervous system, the retina serves to transform light signals into electric pulses [1]. Its development process consists of multistage cell proliferation and differentiation with delicate control of spatiotemporal gene expression [2, 3]. Any perturbation in the gene regulatory network may bring about a cascade effect, disturbing retinal development and inducing visual loss [4]. Knowledge of gene expression regulation is the kernel of the development process, which may aid us in understanding retinal development disorders and degeneration diseases.

Noncoding RNAs (ncRNAs), including miRNAs, circRNAs, and lncRNAs, play vital roles in physiological retinal development, homeostasis, and function [5, 6]. Different classes of ncRNA function differently [2]. miRNAs can regulate gene expression by degrading mRNAs with corresponding miRNA response elements (MREs) [7]. Previous research has investigated their dynamic expression patterns in the developing mouse retina. Torre et al. discovered that three miRNAs, let-7, miR-125, and miR-9, are indispensable for progenitor identity maintenance during retinal development [8, 9]. In contrast, lncRNAs are multifunctional factors in transcriptional and posttranscriptional regulation, such as nuclear topological organizers, chromatin epigenetic state modifiers, or competitive endogenous RNA decoys [10]. Their time-series expression alterations during the development of six ocular tissues have been characterized [11]. Coexpression analysis can successfully infer functional lncRNAs regulating mRNAs. Ren et al. conducted a comprehensive coexpression analysis of lncRNAs and mRNAs in traumatic brain injury patients, providing novel insight for lncRNAs in human neuron injury [12]. Moreover, lncRNAs prominently affect neuron development by sponging miRNAs [13, 14]. In addition, circRNAs are endogenous ncRNAs with a closed-loop structure, which guarantees their high stability [6, 15, 16].. They can sponge miRNAs to alleviate target mRNA degradation in multiple cellular biological processes and diseases, including cancer and neurodegenerative disorders [17]. Jing et al. unravelled the circular transcriptome in the developing mouse retina and highlighted circTULP4 and circHIPK2 as significant miRNA sponges [18]. Recent studies further investigated ncRNAs participating in abnormal retinal development or degenerative diseases. The pathological circRNA profile of retinal neovascularization and chronic ocular hypertension model partially illustrated the pathogenesis [19, 20]. Retinitis pigmentosa is a group of retinal genetic disorders with developmental abnormalities that cause irreversible visual loss [21, 22]. Related investigation profiled miRNAs and lncRNAs in retinal cells under different stress conditions [23–25]. They screened pivotal ncRNAs in retinitis pigmentosa progression and investigated their underlying pathological mechanism. The ncRNA regulation mechanism of physiological retinal development can be a reference to abnormal retinal development and degenerative disease, which may facilitate related research in the future.

However, a time-series transcriptomic analysis integrating circRNA, lncRNA, miRNA, and mRNA profiles in developing the retina has not yet been performed. The crosstalk of multiple ncRNAs in retinal neurogenesis regulation remains poorly elucidated.

Here, we performed whole-transcriptome sequencing of developing mouse retinas in E14.5, P1, P7, P12, P17, and adult mice. The expression profiles of circRNAs, lncRNAs, miRNAs, and mRNAs were comprehensively characterized. Using various bioinformatics tools, we systematically identified key circRNAs, lncRNAs, and miRNAs related to retinal neurogenesis and partially unravelled the underlying regulatory network.

## Results

### Multiclass transcriptomic landscape of retina development

**Fig. 1:**
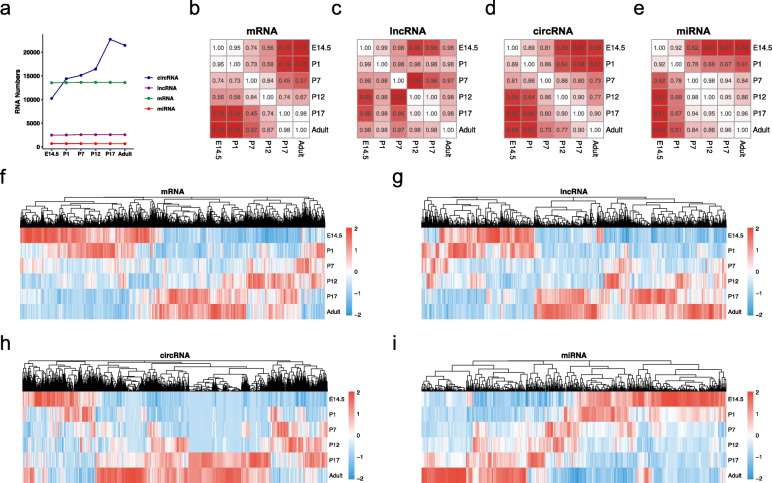
Characteristics of the multiclass transcriptome during retinal development. **(a)** Multiclass RNA numbers identified in six developmental stages. **(b-e)** Canonical correlation heatmap of four transcriptomes in the developing retina. **(f-i)** Heatmap of differentially expressed mRNAs, lncRNAs, circRNAs, and miRNAs identified by time-series analysis

It is acknowledged that the mouse retinal cell types almost develop from E12 to P7. Thus, E14.5, P1, and P7 were selected to represent the primary development stage in this study. To further understand the differentiation and maturation of neuroretinal cells, we have also added three time points P12, P17, and P60 (adult )[26]. At the six developmental time points, we constructed three RNA libraries from the retina to profile four classes of RNA. LncRNA-seq, circRNA-seq and sRNA-seq successfully obtained 100,857,676, 102,423,426 and 12,423,426 reads from the developing retina on average, comprising at least 95.77% of Quality 20 (Q20) reads and 90.51% of Q30 reads. After normalization and filtration, we successfully detected 13,647 mRNAs, 2600 lncRNAs, 27,319 circRNAs, and 704 miRNAs in total. The annotations showed that most lncRNA-seq and circRNA-seq reads could be mapped to coding DNA sequences (CDSs), and miRNAs were the dominant class of small RNAs in small RNA sequencing (Supplementary Fig. 1). The average lengths of the mRNAs, lncRNAs, and circRNAs were 4552, 2915, and 703 nts, respectively. Their similar normalized expression values in six stages supported the subsequent analysis (Supplementary Fig. 1). Next, we evaluated the variability of four RNA developmental profiles by transcript numbers and correlation coefficients. The numbers of circRNAs were significantly increased from 10,234 in E14.5 to 22,703 in adults, while the numbers of mRNAs, lncRNAs and miRNAs remained stable (Fig. 1a). The correlation heatmap further indicated that the mRNA profile had the lowest correlation coefficients during development, followed by circRNAs, with a minimum of 0.55 in E14.5 and P17 (Fig. 1b-e). Through single time-series differential expression analysis, we identified 10,336 mRNAs, 1755 lncRNAs, 17,468 circRNAs, and 649 miRNAs across six time points of development (Fig. 1f-i).

### Identification of key circRNAs related to retinal neurogenesis

**Fig. 2:**
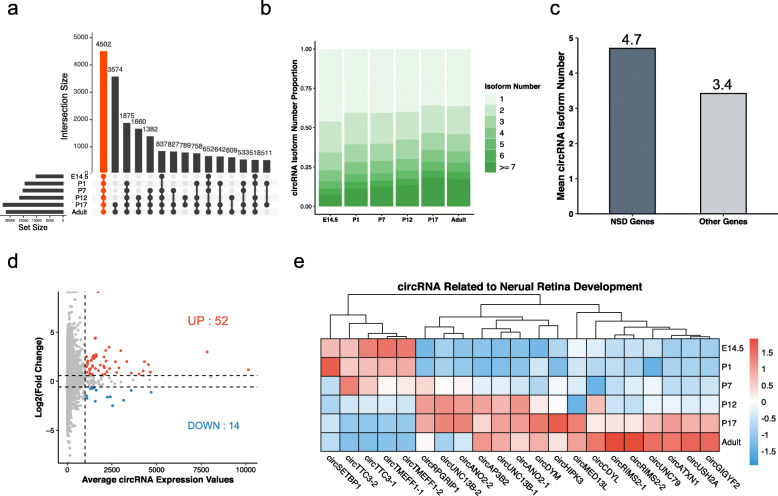
Identification of key circRNAs with cognate nervous system development genes. **(a)** UpSet plot of identified circRNA intersections across six stages. **(b)** circRNA isoform numbers during retinal development. **(c)** Average circRNA isoform numbers of nervous system development genes and other genes. **(d)** Scatterplot of circRNAs filtered by log2(fold change) and average expression values. **(e)** Heatmap of key circRNAs with cognate nervous system development genes

We visualized the intersection of circRNAs in six developing retinas (Fig. 2a). The results revealed that only 4502 circRNAs (16.47%) were constantly expressed, while most mRNAs (98.46%), lncRNAs (93.53), and miRNAs (95.45%) were consistently transcribed during retinal development (Supplementary Fig. 2). The stacked bar plot demonstrated the splicing diversity of circRNAs in the development process, especially in the later stages (Fig. 2b). Approximately 20% of genes could be transcribed and spliced into more than seven circRNA isoforms in the adult retina compared to 10% in the E14.5 retina. To screen the circRNAs with cognate neurogenesis genes, we downloaded the gene list of nervous system development terms (GO:0007399) from Gene Ontology Resource [27]. Splicing isoform analysis revealed that nervous system development (NSD) genes produced more circRNA isoforms than other genes (Fig. 2c). Finally, we screened 66 candidate key circRNAs by the expression threshold and fold change, which included 54 upregulated circRNAs and 14 downregulated circRNAs (Fig. 2d). Integrating with the NSD gene list, we identified 21 circRNAs as key regulators of retinal neurogenesis (Fig. 2e).

### Identification of key lncRNAs related to retinal neurogenesis

**Fig. 3:**
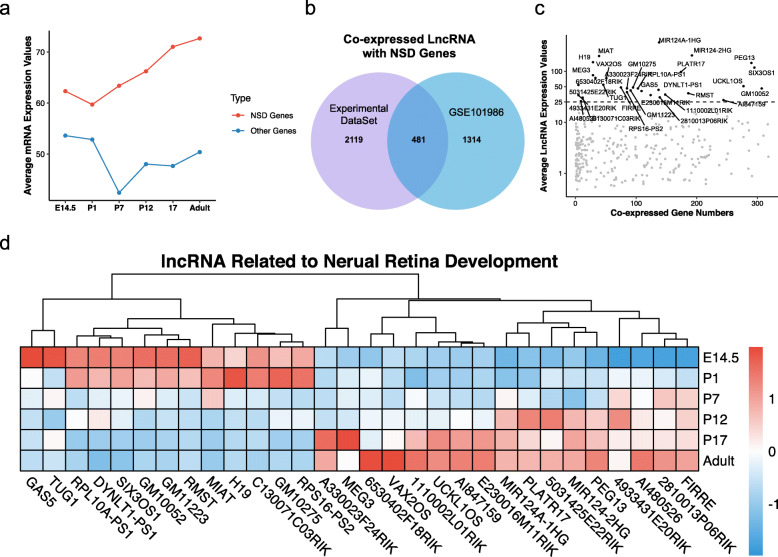
Identification of key coexpressed lncRNAs with nervous system development genes. **(a)** Average mRNA expression values of nervous system development genes and other genes. **(b)** Venn plot of coexpressed lncRNA numbers with nervous system development genes in our experimental dataset and GSE101986. **(c)** Scatter plot and expression filtration of coexpressed lncRNAs. **(d)** Heatmap of key lncRNA expression during retinal development

To investigate neurogenesis-related lncRNAs, we first analysed NSD gene expression patterns in the developing mouse retina. Remarkably, the NSD genes showed higher expression values and increasing patterns during development (Fig. 3a). By coexpression analysis between the differentially expressed lncRNAs and NSD genes in our experimental dataset and GSE101986, we identified 346 coexpressed lncRNAs with NSD genes. Most coexpressed lncRNAs had low expression (RPM < 10). We defined 30 high-abundance coexpressed lncRNAs as regulators of neural retina development with average expression values over 25 RPM (Fig. 3b, c). The heatmap showed that 17 key lncRNAs, including Meg3, Uckl1os, Vax2os, Platr1,7, and Peg13, were upregulated, and 13 key lncRNAs, including Tug1, H19, Miat, and Six3os1, were downregulated during retinal development (Fig. 3d).

### Identification of key miRNAs related to retinal neurogenesis

**Fig. 4:**
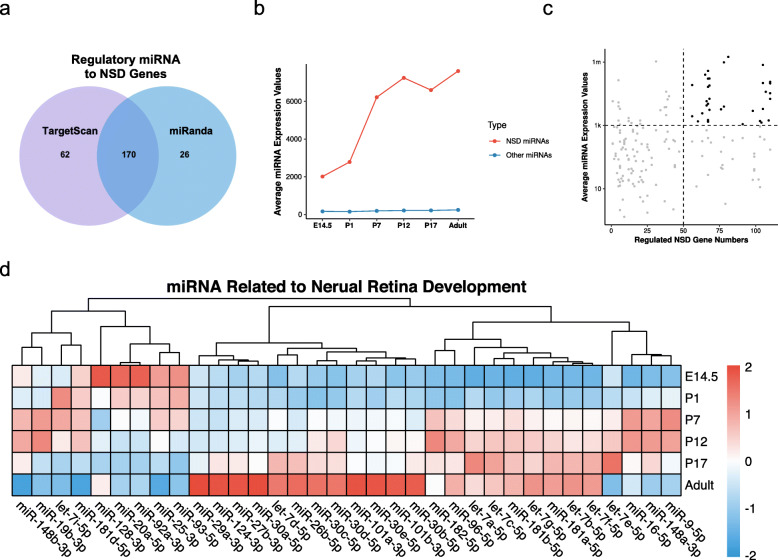
Identification of key miRNAs regulating nervous system development genes. **(a)** Predictive miRNAs regulating nervous system development genes by miRanda and TargetScan. **(b)** Average expression values of nervous system development-related miRNAs and others. **(c)** Key miRNA screening by expression and NSD gene number. **(d)** Heatmap of key miRNAs in developing retina

miRNAs are known to regulate gene expression through miRNA response elements. Here, we predicted the MRE of NSD genes to discover the regulatory miRNAs in retinal neurogenesis. Combining miRanda and TargetScan, 170 miRNAs were discovered to have retinal neurogenesis regulation ability (Fig. 4a). We investigated their expression patterns during retinal development. The 170 miRNAs had more prominent expression levels than the others, and they were significantly upregulated during retinal development (Fig. 4b). Their average expression levels and NSD gene regulation ability were visualized in a scatter plot. With the NSD gene numbers and expression level criteria, we identified 34 miRNAs as retinal neurogenesis regulators (Fig. 4c). Most of the key miRNAs were upregulated during retinal development, including miR-16-5p and miR-29a-3p. Only 9 key miRNAs were downregulated, including miR-128-3p (Fig. 4d).

### Construction of the Core circRNA/lncRNA-miRNA-mRNA Network

**Fig. 5:**
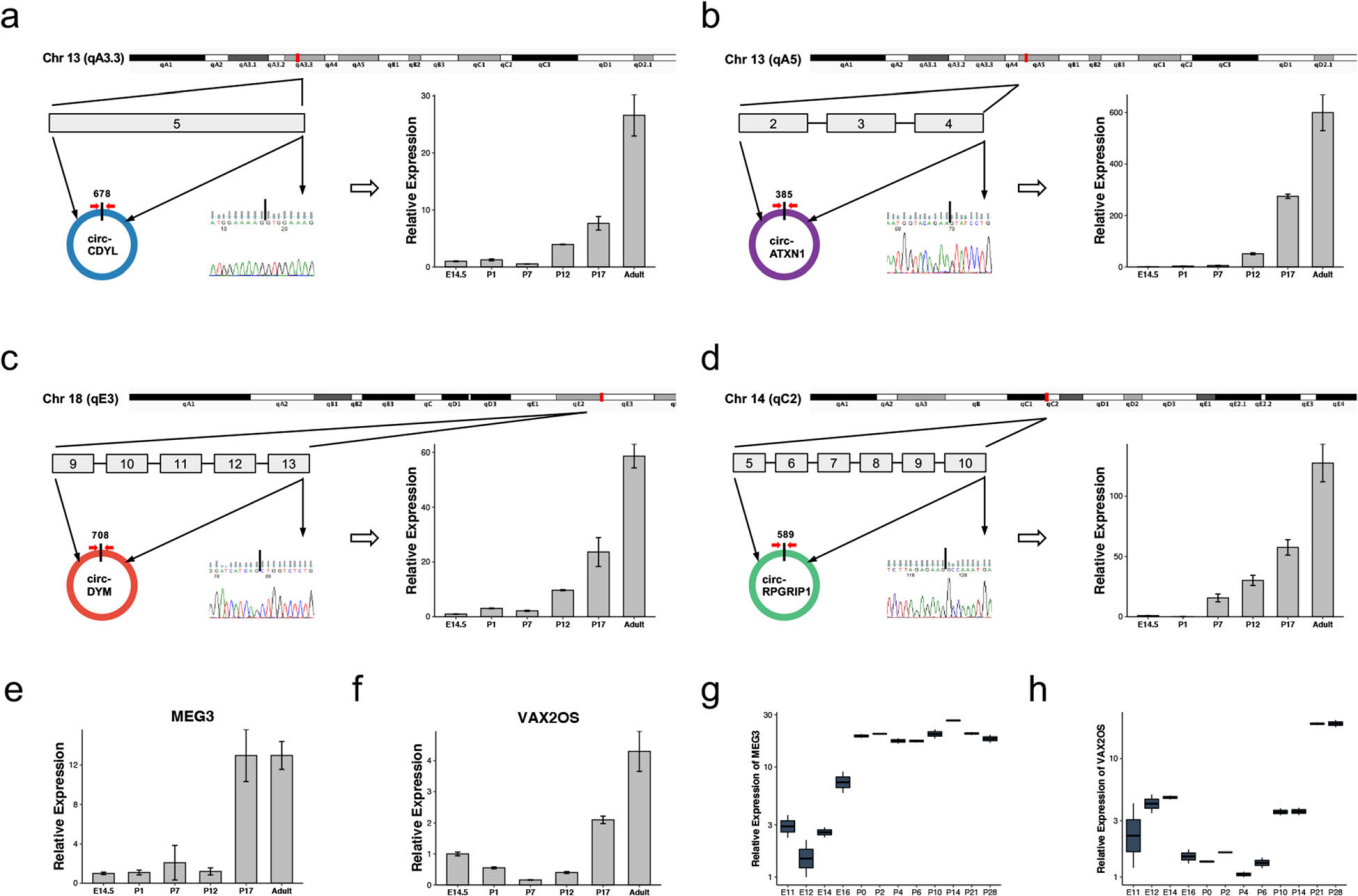
Quantitative real-time PCR validation of 4 key circRNAs and 2 key lncRNAs. **(a-d)** Essential information and validated upregulated expression of circ-CDYL, circ-ATXN1, circ-DYM, and circ-RPGRIP by qRT–PCR with divergent primers designed according to splice junctions. **(e, f)** Increasing expression of lncRNA Meg3 and Vax2os validated by qRT–PCR. **(g, h)** Upregulated expression of lncRNA Meg3 and Vax2os in GSE101986

**Fig. 6:**
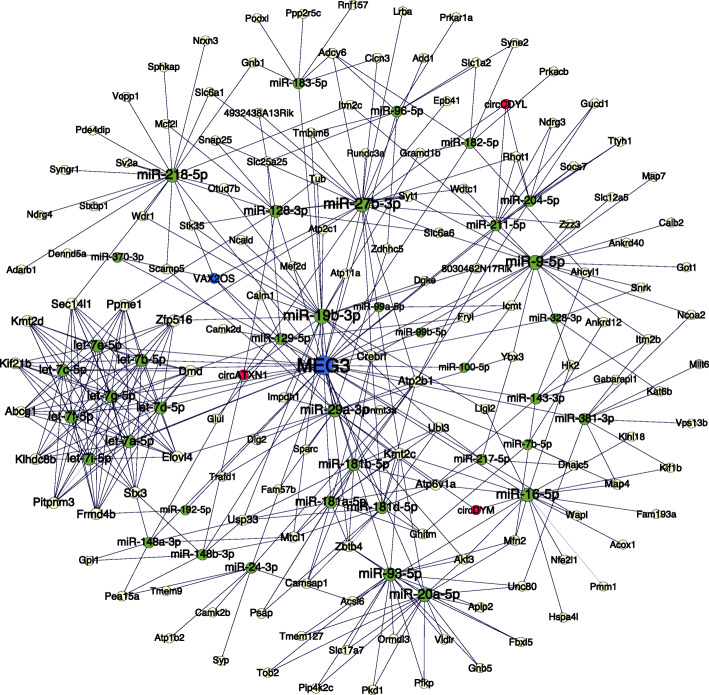
Construction of the regulatory circRNA/lncRNA-miRNA-mRNA network in the developing retina

According to ceRNA theory, circRNAs and lncRNAs could act as miRNA sponges in the developing retina. We hypothesized that the identified key circRNAs and lncRNAs could sponge miRNAs in the developing retina and selected circCDYL, circDYM, circATXN1, circPRGRIP1, lncRNA Meg3, and lncRNA Vax2os for further analysis. Their expression patterns were validated by qRT–PCR. Consistent with our RNA-seq results, circCDYL, circDYM, circATXN1, and circPRGRIP1 were upregulated tens to hundreds of fold during retinal development. They were approximately 385 to 708 nt in length and consisted of 1 to 5 exons from their cognate genes (Fig. 5a-d). The lncRNAs Meg3 and Vax2os showed identical increasing expression patterns in qRT–PCR and GSE101986 (Fig. 5e-h). Their upregulated expression confirmed ceRNA network construction. The regulatory circRNA-miRNA, lncRNA-miRNA, and miRNA-mRNA pairs were determined by the combination of miRanda and TargetScan. After filtration, 39 miRNAs, including miR-16-5p, miR-29a-3p, and miR-128-3p, were sponged by circCDYL, circDYM, circATXN1, lncRNA Meg3, and Vax2os. No miRNAs were sponged by circRPGRIP1 according to our filtration criterion. The 39 identified miRNAs can regulate 135 downstream genes in the developing retina. Taken together, a regulatory network consisting of 3 circRNAs, 2 lncRNAs, 39 miRNAs, and 135 mRNAs was constructed (Fig. 6).

### Functional analysis of circRNA/lncRNA-miRNA-mRNA network

**Fig. 7:**
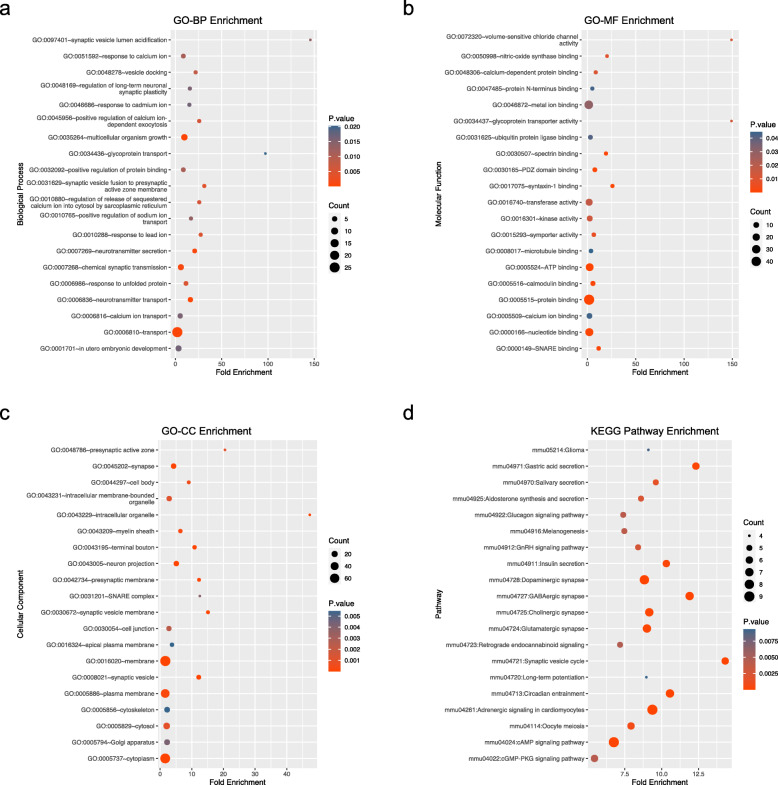
Functional enrichment analysis of genes regulated by key circRNAs and lncRNAs. **(a)** GO biological process enrichment of genes regulated by key ncRNAs. **(b)** GO molecular function enrichment of genes regulated by key ncRNAs. **(c)** GO cellular component enrichment of genes regulated by key ncRNAs. **(d)** KEGG pathway enrichment of genes regulated in the network

Functional enrichment analysis of regulated mRNAs suggested that these genes were enriched in 53 Gene Ontology Biological Process (GO-BP) terms, 26 Molecular Function (GO-MF) terms, and 55 Cellular Component (GO-CC) terms. Figure 7 displays the top 20 pathways or GO terms enriched by regulated differentially expressed mRNAs. From the results, we found that these genes were mainly involved in GO:0007268 ~ chemical synaptic transmission, GO:0035264 ~ multicellular organism growth, and GO:0050998 ~ nitric-oxide synthase binding (Fig. 7a, b). The genes were significantly enriched in GO:0045202 ~ synapse, GO:0043005 ~ neuron projection, and GO:0008021 ~ synaptic vesicle (Fig. 7c). Furthermore, Kyoto Encyclopedia of Genes and Genomes (KEGG) enrichment analysis was performed based on the genes involved in the circRNA/lncRNA-miRNA-mRNA, and the results were visualized in a bubble diagram (Fig. 7d). The results showed that these genes were significantly enriched in glioma, synaptic vesicle cycle, and various synapses.

## Discussion

The vertebrate retina is a highly evolved and sophisticated biological organ [28, 29]. Its layered structure and diverse cell composition originate from multipotent neural progenitors [1]. Such a transition is driven by the concerted control of endogenous regulators, including ncRNAs, whereas the dissection of multiclass RNA crosstalk in retinal neuron development remains challenging [4]. Recently, several studies confirmed the correlated roles of neuroprotective pathways in normal neurodevelopment and neurodegenerative conditions [30, 31]. Thus, understanding gene expression regulation in retinal development contributes to the exploitation of therapy for retinal neurodegenerative disorders. Herein, we profiled circRNAs, lncRNAs, miRNAs, and mRNAs in the developing mouse retina and focused on ncRNAs related to retinal neurogenesis.

To our knowledge, this is the first time-series analysis integrating mRNA, lncRNA, circRNA, and miRNA profiles in the developing mouse retina. Six time points from E14.5 to adulthood. We identified 13,647 mRNAs, 2600 lncRNAs, 27,319 circRNAs, and 704 miRNAs from the developing retina in total. A previous study probed 9029 circRNAs in the mouse retina [18]. In contrast, we used RNase R to digest linear RNAs in circRNA library construction, tremendously enlarging our landscape of circRNAs in the developing retina. The circRNA number increased drastically during development, from 10,234 in E14.5 to 22,703 in adults, while the numbers of mRNAs, lncRNAs and miRNAs were roughly stable. Considering the low circRNA profile correlation coefficients in ncRNAs, circRNAs are the most variable ncRNAs in the developing retina. We also intersected the circRNAs of different time points and found that only 16.47% of circRNAs were constantly expressed in six stages. The increasing splicing diversity of circRNA isoforms during development agrees with the increasingly complex cell composition in the retina. Overall, these results suggested that circRNAs are highly variable regulators in the retinal development process.

Multiple classes of RNA profiles over time facilitate the prediction of ncRNA regulatory effects in the developing retina. We used different strategies to screen key ncRNAs according to their characteristics. For circRNAs, NSD genes have more spliced circRNA isoforms. We also intersected the circRNA profiles with the NSD gene list. As a result, 21 circRNAs with high abundance and differential expression were screened, including circTTC3–1 and circTTC3–2. Among these circRNAs, circSETBP1, circTTC3, circDYM, circHIPK3, circMEDBL, circCDYL, circRIMS2, and circATXN1 have been reported in previous studies. Yao validated that circDYM abated depressive disorder by sponging miR-9 to regulate microglial activation, and circATXN1 can regulate glioma angiogenesis via the miR-526b-3p/MMP2 pathway [32, 33]. Their proven roles in the central nervous system suggested that they may also act as miRNA sponges in retinal development or degenerative disorders. The other key circRNAs were first detected by RNA sequencing. Interestingly, we filtered circANO2 and circUSH2A, whose host genes are associated with retinal development. Ano2 is a calcium-dependent chloride channel protein that cooperates with a presynaptic protein complex to mediate light perception amplification in photoreceptor terminals [34]. Mutations within Ush2a are the most common cause of retinitis pigmentosa and Usher syndrome type 2[35, 36]. Their circular transcripts may also be implicated in retinal nervous system development. Next, we characterized the expression patterns of NSD genes in the developing retina and successfully identified 30 key coexpressed lncRNAs, including Gas5, Tug1, Rmst, Miat, Meg3, Vax2os, and Peg13. Several identified lncRNAs have been reported in retinal development studies. For example, Tug1 is associated with normal photoreceptor development, contributing to rod differentiation and survival. Loss of Tug1 in the neonatal retina can cause photoreceptor outer segment structural defects, enhanced apoptosis, and expression changes of key transcription factors [37]. Meg3 was upregulated in the light-stimulated retina, and the silencing of Meg3 ameliorated light-induced retinal damage both in vivo and in vitro. Our findings indicated the underlying regulatory mechanism induced by lncRNAs in the development process [38]. Additionally, we inferred the miRNA-binding elements of nervous system development genes and classified the miRNAs into NSD-related miRNAs and other miRNAs. The extraordinarily high expression of NSD-related miRNAs supported their irreplaceable property in development regulation. Several key miRNAs that control retinal development have been functionally described, such as miR-182, miR-96, and miR-181 [39, 40].

Considering the average expression level and variability, we selected four key circRNAs and two key lncRNAs for analysis by qRT–PCR. Their increasing expression during retinal development was confirmed, and their tens- or hundreds-fold upregulated expression levels suggested that they may participate as ceRNAs in retinal development regulation. Thus, we predicted the RNA-RNA regulatory pairs and constructed a circRNA/lncRNA-miRNA-mRNA network based on competitive endogenous RNA theory. After filtration, a competitive endogenous RNA network consisting of 3 circRNAs, 2 lncRNAs, 39 miRNAs, and 135 mRNAs was determined. Several miRNAs in the network are known as regulators of retinal development, such as miR-181 and miR-96/182/183. miR-128, a brain-enriched miRNA, is implicated in the control of neurogenesis and synaptogenesis [41]. Here, it is discovered to be sponged by circATXN1. In addition, miR-204 and miR-211 have only one nucleotide difference in their mature mouse sequences and share an identical seed sequence [2]. Knockdown of miR-211 activity in mice led to chronic photoreceptor dystrophy, and miR-204 contributes to axon guidance in the retina [42]. The increasing expression of circCDYL can alleviate the degradation effect of miR-204 and miR-211 to target mRNAs. Within the network, we also noticed that lncRNA Meg3 collectively sponged the miRNA let-7 family, which regulates the neurogenic potential of Müller glia and plays an essential role in retinal regeneration [43]. The lncRNA Meg3 is the hub ceRNA in the circRNA/lncRNA-miRNA-mRNA network, with 39 sponged miRNAs in total, possibly due to its extraordinary length of 9701 nt. Previous studies have validated the function of Meg3 related to its methylation and the PI3K/Akt/mTOR signalling pathway in diabetic retinopathy [44]. Our analysis revealed the vast potential of lncRNA Meg3 as a ceRNA in retinal development.

To summarize the main pathways of the circRNA/lncRNA-miRNA-mRNA network, we performed functional enrichment analysis in Gene Ontology and KEGG. Regulated genes are primarily associated with synaptic signalling and nervous system development. Related pathways or terms included neurotransmitter transport, metal ion binding, neuron projection, and synaptic vesicle cycle.

Although we discovered key neurogenesis-related ncRNAs by whole-transcriptome sequencing and bioinformatics analysis, further experimental verification is still needed in the future.

## Conclusions

We profiled circRNA, lncRNA, miRNA, and mRNA expression in E14.5, P1, P7, P12, P17, and adult mouse retinas by whole-transcriptome sequencing, and the expression patterns of circRNAs, lncRNAs, miRNAs, and mRNAs were characterized. CircRNAs showed the highest variability compared to the other ncRNAs. The key circRNAs, lncRNAs, and miRNAs regulating retinal neurogenesis were screened through different strategies. We performed qRT–PCR of six key ncRNAs to validate their increasing expression. Functional analysis suggested that they were involved in related neuron development pathways through the circRNA/lncRNA-miRNA-mRNA network.

## Methods

### Animals

C57BL/6 J mice were obtained from Vital River Laboratory (Beijing, China), kept in a pathogen-controlled environment in standard cages and fed ad libitum. All animal experiments were performed strictly according to the ARVO Statement for the Use of Animals in Ophthalmic and Vision Research and were approved by the Animal Care and Use Committee of Wenzhou Medical University.

### RNA extraction

Mice received general anesthesia by intraperitoneal injection with the mixture of ketamine (85 mg/kg) and xylazine (5 mg/kg). The ocular tissues were topically anesthetized by proparacaine hydrochloride (Alcon, Fort Worth, TX, USA). Retinas were dissected from mice in six developmental periods: embryonic day (E) 14.5, postnatal day (P) 1, 7, 12, 17, and adult (8 weeks). Briefly, the mice were euthanized, and retinas were isolated from eyes and moved into RNA-Solv Reagent (Omega Bio-Tek, Norcross, GA, USA). A T10 basic S25 ULTRA-TURRAX Disperser (IKA, China) was used to homogenize retinas. According to the manufacturer’s instructions, total RNA was extracted using a miRNA Kit (Omega Bio-Tek, Norcross, GA, USA). RNA quantity was assessed using an Agilent 2100 Bioanalyser (Santa Clara, CA, USA).

### Library construction and whole-transcriptome sequencing

Three RNA libraries were constructed individually to fully characterize the complex transcriptome of the developing retina. Libraries of lncRNAs were prepared after removing rRNA using the Ribo-ZeroTM kit (Epicentre, Madison, WI, USA) and were submitted for 150-bp paired-end sequencing using the Illumina HiSeq 3000 platform. Similarly, sRNA libraries were sequenced on the Illumina HiSeq 2500 sequencing platform at RiboBio (Guangzhou, China). For the circRNA library, the rRNAs were depleted from total RNA using the Ribo-ZeroTM kit. Then, RNA was treated with RNase R (Epicentre, Madison, WI, USA) to remove linear transcripts and was then fragmented to approximately 200 bp. The purified RNA fragments were subjected to first- and second-strand cDNA synthesis following adaptor ligation according to the NEB Next Ultra™ RNA Library Prep Kit manual for Illumina (NEB, USA). The purified library products were evaluated using the Agilent 2200 TapeStation and Qubit®2.0 (Life Technologies, USA) and were sequenced on a HiSeq 3000 in paired-end 150-bp mode.

### Data pre-processing and transcriptomic analysis

FastQC was performed for quality control of the raw data, and paired-end reads were trimmed using Trimmomatic [45]. The ribosomal RNAs were removed according to the RNA central database, and clean data were obtained [46]. Next, lncRNA reads were mapped to the reference genome mm10 using Hisat2 [47]. HTSeq was used to count the read numbers mapped to each gene [48]. BWA aligned the sRNA library reads, and miRNAs were identified using miRDeep2 [49, 50]. For the circular transcriptome, two algorithms, CIRI2 and CIRCexplorer2, were used to detect circRNAs [51, 52]. The circRNAs that were detected by both methods were considered identified circRNAs. The expression values of lncRNAs, mRNAs, circRNAs, and miRNAs were normalized by reads per million (RPM) in edgeR [53]. We retained the transcripts that were significantly detected at more than two time points (RPM > 1). Then, single time-series differential expression analysis by maSigPro was performed to identify differentially expressed RNAs [54]. False discovery rate correction was used in the analysis. FDR-corrected *P* values < 0.05 were considered significant.

### Retinal neurogenesis-related circRNA identification

First, the intersection plot of circular transcripts in six stages was visualized in the UpSet package in Bioconductor. Based on circRNA annotation in CIRI2 and CIRCexplorer2, the cognate circRNA isoforms were investigated in different stages. We next downloaded the development genes annotated by nervous system development (GO Accession: 0007399) from Gene Ontology Resource to examine whether the development-related genes had more circRNA splicing isoforms [27]. Then, we screened the circRNAs with fold change > 1.5 and average expression value > 1000 to find potential key circRNAs. Finally, the key circRNAs associated with the nervous system development process were visualized in a heatmap.

### Retinal neurogenesis-related lncRNA identification

To investigate the underlying key lncRNAs in neural retina development, we performed coexpression analysis by calculating Pearson’s correlation coefficients between nervous system development genes and differentially expressed lncRNAs. We combined our RNA-seq dataset with the former dataset from Gene Expression Omnibus to make the coexpression inference reproducible (Accession: GSE101986, [55]). The lncRNAs highly correlated with the development genes (coefficient > 0.8) in two datasets were considered nervous system development-related lncRNAs. Last, the lncRNAs with substantial abundance in our experiment (average RPM > 25) and significant correlation with nervous system development genes were defined as key lncRNA regulators of neural retina development.

### Retinal neurogenesis-related miRNA identification

The key miRNAs were defined as critical regulators of the nervous system development process. The miRNA-gene predictive analysis of the differentially expressed miRNAs was performed using miRanda (http://cbio.mskcc.org/miRNA2003/miranda.html) and TargetScan (http://www.targetscan.org/) through miRNA response elements in NSD genes [56, 57]. The software was executed in default mode, and the intersecting NSD genes were preserved. miRNAs possessing high expression (average RPM > 1000) and substantial NSD gene regulation capability (regulated genes > 50) were regarded as key miRNA regulators in neural retina development.

### Quantitative real-time PCR validation of key ncRNAs

Four differentially expressed circRNAs were confirmed by qRT–PCR validation. The primers were designed based on the back-spliced sequences. First, total RNA was reverse transcribed into cDNA using the Geneseed II First Strand cDNA Synthesis Kit (Geneseed, China) in a 20-μl reaction volume. Second, real-time PCR was performed on an Applied Biosystems 7500 Real-time PCR Analyser (Applied Biosystems, USA) using Geneseed qPCR SYBR Green Master Mix (Geneseed, China). The reaction conditions were as follows: initial incubation at 95 °C for 5 min, followed by 40 cycles of 10 s of denaturation at 95 °C, 34 s of annealing, and extension at 60 °C. The average Ct value was used to calculate the relative expression of circRNA through the comparative 2-△△Ct method.

For lncRNA Meg3 and lncRNA Vax2os, their conserved sequences were obtained from NCBI (National Center of Biotechnology Information). We designed primers that were then synthesized by RiboBio (Guangzhou, China). For each sample, total RNA was reverse transcribed into cDNA using a Geneseed II First Strand cDNA Synthesis Kit (Geneseed, China). Real-time PCR was carried out on a QuantStudio 5 Real-Time PCR System (Applied Biosystems, Foster City, CA, USA) using 2× SYBR Green Mix. The relative levels of target genes were calculated using the 2-△△Ct method using GAPDH as the reference gene. The primer sequences used in this study are listed in Supplementary Table 1.

### circRNA/lncRNA-miRNA-mRNA network construction

To construct a circRNA/lncRNA-miRNA-mRNA axis, we started with the validated circCDYL, circDYM, circATXN1, lncRNA Meg3, and lncRNA Vax2os. Based on the sequences of miRNA response elements, target RNAs of miRNAs were determined by the most common software programs for miRNA target prediction, miRanda (http://cbio.mskcc.org/miRNA2003/miranda.html) and TargetScan (http://www.targetscan.org/) [56, 57]. The software was executed in default mode, and the intersected target RNAs were preserved. The target miRNAs and mRNAs with differential expression and high abundance (mRNA average RPM > 100, miRNA average RPM > 1000) were screened. For mRNA, only those with upregulated expression at the later time points (fold change > 1.5) were retained according to the ceRNA theory. Then, Gephi software (https://gephi.org/) was used to construct the regulatory network.

### Functional enrichment analysis

Based on the regulatory network, the role of the identified key noncoding RNAs was analysed according to the functions of their regulated genes. These genes were submitted to Gene Ontology (GO) analysis to identify enrichments in biological process, molecular function, and cellular component terms [27]. Via two-sided Fisher’s exact test with false discovery rate correction, we calculated the *P* value of each term, and those with *P <* 0.05 were considered statistically significant. Kyoto Encyclopedia of Genes and Genomes (KEGG) pathway analysis was performed in DAVID Function Annotation Tool 6.8 with the criterion of FDR-corrected *P <* 0.0 5[58]. The terms with minimum *P* values were selected and visualized.

### Statistical analysis

Statistical analyses between two groups were analysed by Student’s t-test in R. Statistical tests were all two-tailed, and *P <* 0.05 was considered significant. Quantitative real-time PCR in each group was performed in three replicates, and data are expressed as the mean ± SEM.

## Supplementary Information

**Additional file 1: Supplementary Figure 1.** Whole-transcriptome sequencing data summary. **Supplementary Figure 2.** Upset plot of intersected transcripts in six stages.

**Additional file 2: Supplementary Table 1.** primer sequences of four circRNAs and lncRNAs in Quantitative Real-time PCR. **Supplementary Table S2.** RNA-RNA pairs in circRNA/lncRNA-miRNA-mRNA Network.

## Data Availability

The datasets generated and analysed during the current study are available in the NCBI Gene Expression Omnibus (GEO) repository under accession number GSE168093 (https://www.ncbi.nlm.nih.gov/geo/query/acc.cgi?acc= GSE168093).

## Abbreviations

PCR: Polymerase chain reaction
ARVO: Association for Research in Vision and Ophthalmology
sRNA: Small RNA
miRNA: Micro RNA
circRNA: Circular RNA
lncRNA: Long non-coding RNA
MRE: miRNA response element
NSD: Nervous system development
ceRNA: Competitive endogenous RNA
GO: Gene Ontology Resource
KEGG: Kyoto Encyclopedia of Genes and Genomes
GEO: Gene Expression Omnibus
