# Supplementary material for: Whole transcriptome sequencing identifies key circRNAs, lncRNAs, and miRNAs regulating neurogenesis in developing mouse retina
Source: BMC Genomics. 2021 Oct 30;22:779. doi: 10.1186/s12864-021-08078-z (PMC8557489; doi:10.1186/s12864-021-08078-z)
Supplement: Supplementary file 1 — Additional file 1: Supplementary Figure 1. Whole-transcriptome sequencing data summary. Supplementary Figure 2. Upset plot of intersected transcripts in six stages. [file 12864_2021_8078_MOESM1_ESM.pdf]

Supplemental Figures

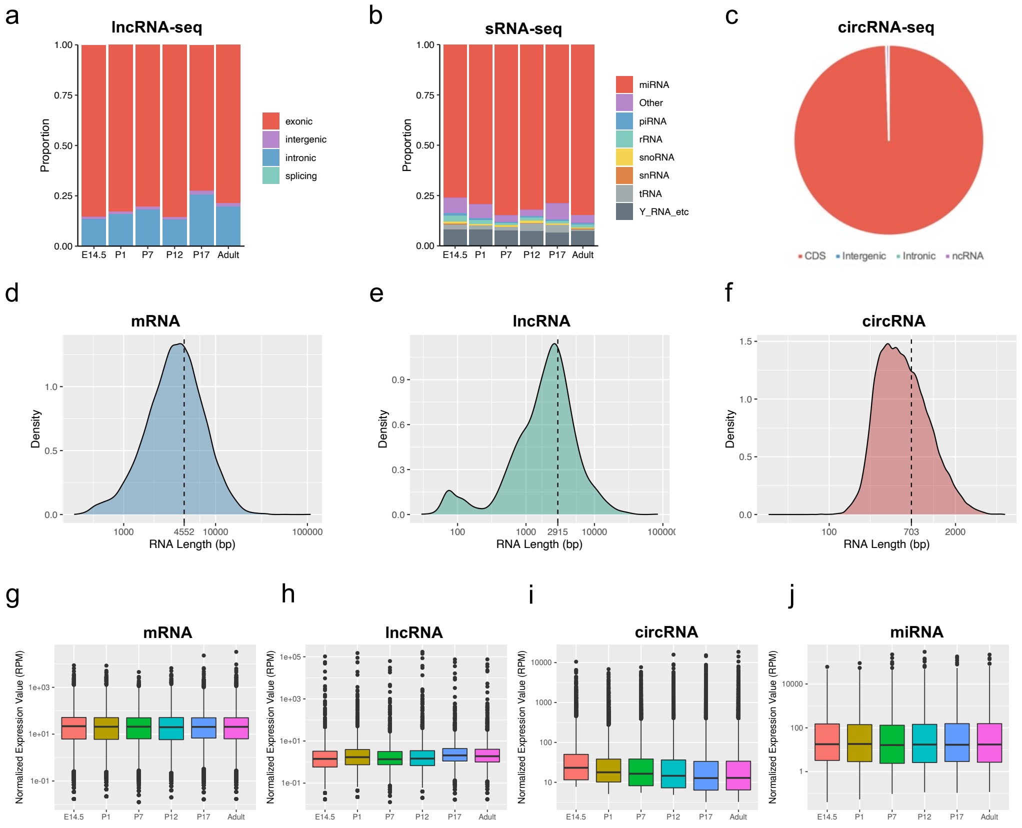

Supplementary Figure 1. Whole-transcriptome sequencing data summary. (a-c) Transcripts annotation from lncRNA-seq, sRNA-seq and circRNA-seq. **(d-f)** Length density plot of mRNA, lncRNA and circRNA. **(g-j)** Expression abundance of four class of transcriptomes across six developmental stages.

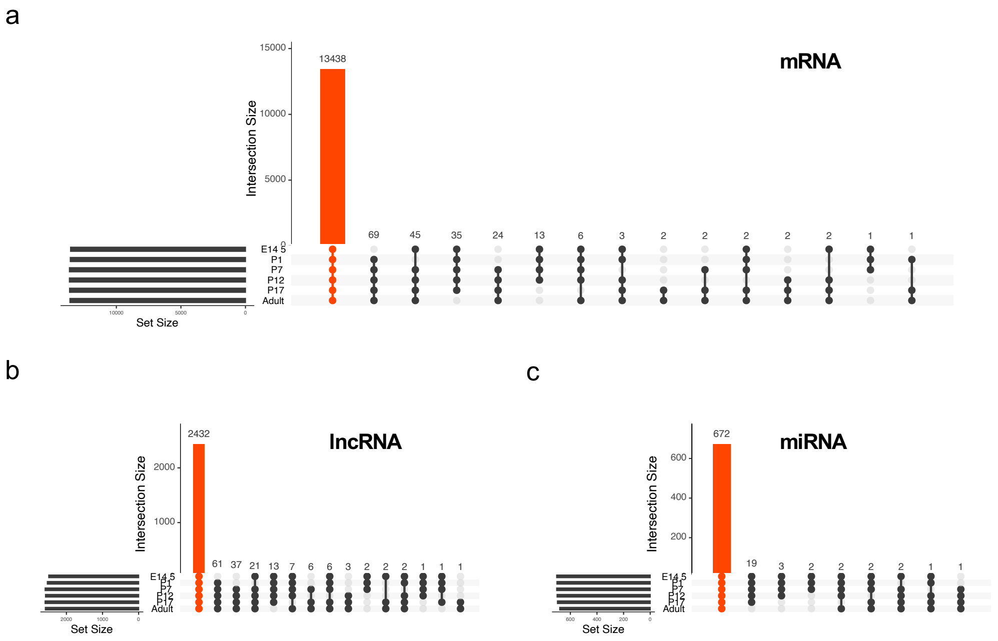

Supplementary Figure 2. Upset plot of intersected transcripts in six stages. (a-c) Overlapped lncRNA, mRNA and miRNA numbers in different stages.
